# Transcriptional changes associated with advancing stages of heart failure underlie atrial and ventricular arrhythmogenesis

**DOI:** 10.1371/journal.pone.0216928

**Published:** 2019-05-13

**Authors:** Mariana A. Argenziano, Michael Xavier Doss, Megan Tabler, Agapios Sachinidis, Charles Antzelevitch

**Affiliations:** 1 Children’s Hospital of Philadelphia, Department of Genetics, Philadelphia, Pennsylvania, United States of America; 2 Lankenau Institute for Medical Research, Wynnewood, Pennsylvania, United States of America; 3 University of Cologne, Institute of Neurophysiology and Center for Molecular Medicine Cologne (CMMC), Cologne, Germany; 4 Lankenau Heart Institute, Wynnewood, Pennsylvania, United States of America; 5 Sidney Kimmel Medical College of Thomas Jefferson University, Philadelphia, Pennsylvania, United States of America; University of Minnesota, UNITED STATES

## Abstract

**Background:**

Heart failure (HF) is a leading cause of mortality and is associated with cardiac remodeling. Vulnerability to atrial fibrillation (AF) has been shown to be greater in the early stages of HF, whereas ventricular tachycardia/fibrillation develop during late stages. Here, we explore changes in gene expression that underlie the differential development of fibrosis and structural alterations that predispose to atrial and ventricular arrhythmias.

**Objective:**

To study transcriptomic changes associated with the development of cardiac arrhythmias in early and late stages of heart failure.

**Methods:**

Dogs were tachy-paced from right ventricle (RV) for 2–3 or 5–6 weeks (early and late HF). We performed transcriptomic analysis of right atria (RA) and RV isolated from control dogs and those in early and late HF. Transcripts with mean relative log2-fold change ≥2 were included in the differential analysis with significance threshold adjusted to p<0.05.

**Results:**

Early HF remodeling was more prominent in RA with enrichment of extracellular matrix, circulatory system, wound healing and immune response pathways; many of these processes were not present in RA in late HF. RV showed no signs of remodeling in early HF but enrichment of extracellular matrix and wound healing in late HF.

**Conclusion:**

Our transcriptomic data indicate significant fibrosis-associated transcriptional changes in RA in early HF and in RV in late HF, with strong atrial predominance. These alterations in gene expression are consistent with the development of arrhythmogenesis in atria in early but not late HF and in the ventricle in late but not early HF.

## Introduction

Heart failure (HF) is a leading cause of hospital admission, affecting an estimated 6.5 million Americans. Projections show that the prevalence of HF will increase 46% by 2030 [1]. Atrial fibrillation (AF) develops in 5%-50% of patients with HF, and its prevalence increases with worsening functional severity of HF [2]. The development of AF in HF appears to be a multifactorial process, including early atrial enlargement and important structural, electrophysiological, biochemical and molecular remodeling [3,4]. Recent studies from our group showed that vulnerability to AF is greater in early vs. late stages of HF (78% vs. 38%). In contrast, ventricular tachycardia/fibrillation (VT/VF) can be more readily induced in late vs. early HF (38% vs. 0%) [5]. In that study, the dimensions of atria and ventricles showed significant changes in early HF, with a relatively small further change in the late stage of HF. Fibrosis developed progressively throughout the 5–6 weeks period of ventricular tachy-paced (VTP), to a much greater extent in atria than in the ventricles. Surface ECG showed major changes including acceleration of heart rate, prolongation of the QRS and QTc, and a significant increase of P wave amplitude in animals with HF.

The VTP model of HF has been shown to mimic the fundamental mechanical, electrophysiological, biochemical and molecular changes observed in human HF [6]. Although this model has been previously used to describe gene expression changes in early stages of HF at 24 hours and 2 weeks in the left atrium (LA) and left ventricle (LV) and in left and right ventricles of normal and VTP dogs at the end stages of HF [7,8], changes in right atrium (RA) in early (2 weeks) and late (5 weeks) stages of HF remain unexplored.

The present study was designed to close this gap of knowledge by performing a transcriptomic analysis using RNA isolated from our previously reported study in which we characterized the electrophysiological, echocardiographic and histological changes associated with early and late stages of HF in a canine ventricular-tachypaced model of HF. The primary objective of this study was to elucidate the transcriptional changes in the right chambers associated with early and late stages of HF in a canine model [5].

We hypothesized that chamber-specific changes in gene expression underlie the differential development of fibrosis and structural alterations that predispose to atrial and ventricular arrhythmias.

## Methods

### Heart failure model: VTP protocol

Our study conforms to the Guide for the Care and Use of Laboratory Animals and was approved by the Institutional Animal Use and Care Committee of the Masonic Medical Research Laboratory. This study is an extension of our previously reported study in which we demonstrated recapitulation of the electrophysiologic, echocardiographic, histologic and arrhythmic manifestations of clinical HF [5].

To produce “early” and “late” stages of HF, adult mongrel or beagle dogs were continuously paced using a modified Medtronic (Minneapolis, Minnesota) pacemaker for a period of 2–3 and 5–6 weeks, respectively. The bipolar pacing electrode was positioned in the interventricular septum of the RV with the aid of fluoroscopy and trans-esophageal echocardiography. After recovering from the procedure (1 day), the dogs were paced at 200–240 bpm. Pulse rate was monitored daily, and a 12-lead electrocardiogram (ECG) was recorded weekly to ensure proper pacing.

The present study, included 3 control dogs, 3 dogs with early HF and 3 dogs with late HF [5]. After euthanasia, a small piece of tissue was isolated from each chamber and snap frozen for RNA isolation and subsequent transcriptomic characterization prior to electrophysiologic study [5].

### Microarray

We evaluated the transcriptome in RA and RV of canine hearts in early HF, late HF or control using 3 biological replicates. Total RNA was extracted from tissue samples using RNeasy Mini Kit with on-column DNase I digestion as recommended by the manufacturer’s instruction (Qiagen, Valencia, California). All reagents and instrumentation pertaining to oligonucleotide microarrays were procured from Affymetrix (Thermo Fisher Scientific, Santa Clara, California). Total RNA (100 ng) was used for amplification and *in-vitro* transcription using the GeneChip 3’ IVT Express Kit as per the manufacturer’s instructions (Thermo Fisher Scientific). The amplified RNA was purified with magnetic beads, and 15 μg Biotin-aRNA was fragmented with fragmentation reagent. 12.5 μg fragmented aRNA was hybridized to Thermo Fisher Scientific Canine Genome 2.0 arrays along with hybridization cocktail solution and then placed in GeneChip Hybridization Oven 645 (Thermo Fisher Scientific) rotating at 60 rpm at 45°C for 16 h. After incubation arrays were washed on GeneChip Fluidics Station 450 (Thermo Fisher Scientific), they were stained with Thermo Fisher Scientific HWS kit as per manufacturer’s protocols. The chips were scanned with Thermo Fisher Scientific GeneChip Scanner 30007G, and the quality control matrices were confirmed with Thermo Fisher Scientific GCOS software following the manufacturer’s guidelines.

#### Identification of differentially expressed genes (DEG)

Background correction, summarization and normalization were done with robust multiarray analysis. The raw dataset was normalized with Quantile normalization method executable with R (Affy)-package carried out at probe feature level. Probe sets that were detected to be present were selected, and those that were absent were eliminated. DEGs were described by a linear model implementing R-LIMMA packages (Linear Models) for microarray data [9,10].

Unsupervised hierarchical clustering of all detected genes was performed using the Euclidean distance and complete linkage method. Volcano plots were performed using R software. DEGs were obtained by Advaita Bio’s iPathwayGuide (http://www.advaitabio.com/ipathwayguide, Advaita Corp., Plymouth, Michigan) using a threshold of 0.05 for statistical significance (p-value) and a log fold change of expression with absolute value of at least 2. The p-value was corrected for multiple comparisons using FDR and Bonferroni.

Pathway and ontology data were analyzed in the context of pathways obtained from the Kyoto Encyclopedia of Genes and Genomes (KEGG) database (Release 81.0+/01-20, Jan 17) [11,12], gene ontologies from the Gene Ontology Consortium database (2016-Sep26) [13], and iPathwayGuide using the proposed Elim and Weight Pruning [14].

### Real time PCR validation

Real-time RT-PCR for 7 selected genes was used to confirm the microarray data. Primers were designed using the UCSC genome (https://genome.ucsc.edu/) database and Primer Blast free access design tool (https://www.ncbi.nlm.nih.gov/tools/primer-blast/). Total RNA was extracted using TRIzol Reagent (Invitrogen, Carlsbad, California) and Qiagen RNeasy (Qiagen, Valencia, California) column. Two micrograms of RNA were used to obtain cDNA using SuperScript IV reverse transcriptase following the manufacturer’s instructions (Thermo Fisher Scientific, Carlsbad, California). Real time PCR analyses were conducted with an Eppendorf Realplex (Eppendorf, Mt Laurel, New Jersey) system using Glyceraldehyde 3-phosphate dehydrogenase (GAPDH) as an endogenous control. The analysis was performed following the ΔΔCt method [15].

## Results

### Global gene expression analysis at early and late stages of HF

**Fig 1:**
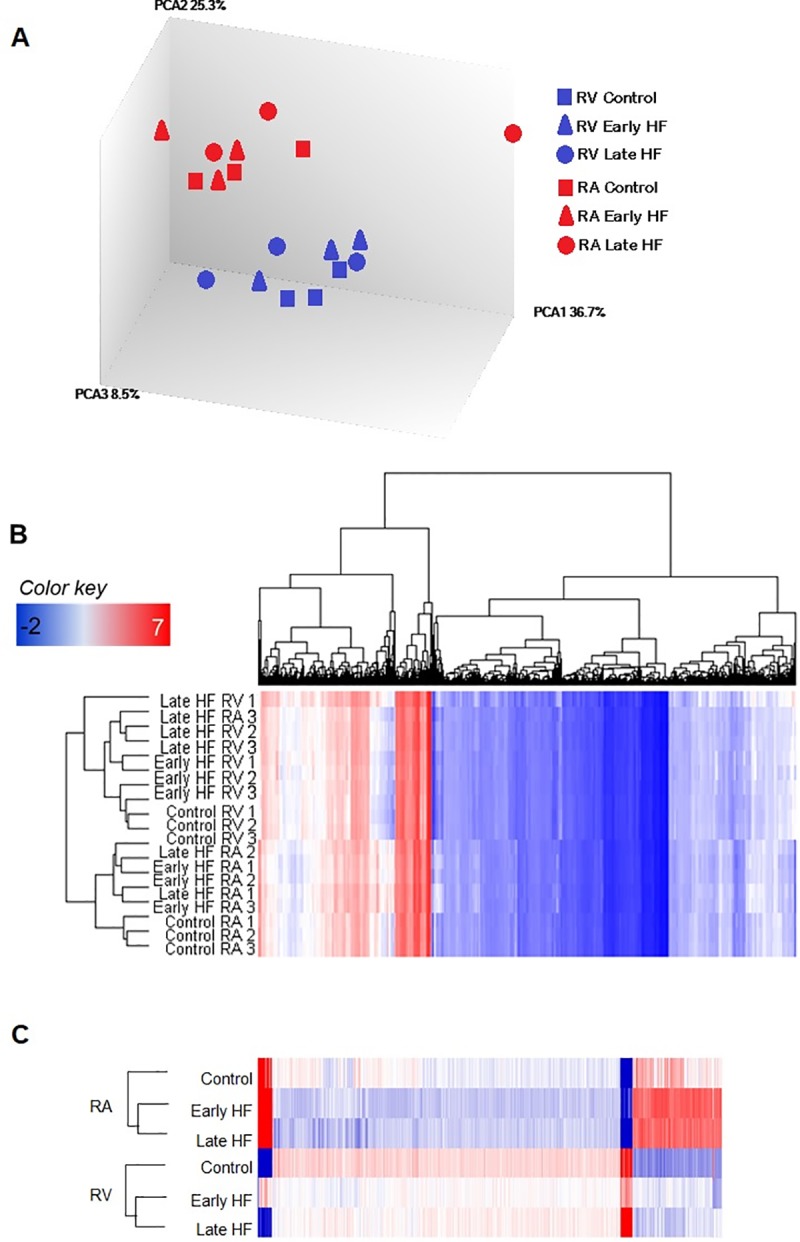
Principal component and clustering show a clear separation between controls and heart failure. (A) Three-dimensional principal component analysis (PCA) plot of multi-array average–normalized data for early and late stages of HF in RA (red) and RV (blue). (B) Unsupervised hierarchical clustering of the global gene expression data. (C) Unsupervised hierarchical clustering of the 534 identified differentially expressed genes (DEGs) for the averaged data. (ANOVA, p<0.01, n = 3). Data are log transformed and normalized. Upregulated genes are displayed in red and downregulated genes in blue. Dendrogram provides a measure of the relatedness of gene expression in each sample (at left) and for each gene (at top).

We examined the transcriptional profiles of RA and RV during early and late stages of HF. The surface ECG parameters measured for control and HF dogs is shown in **Table 1**. Heart samples were a mixed population of cardiomyocytes, fibroblasts and endothelial tissue. To compare the variability between all samples, we performed gene expression cluster analysis. Individual sample clustering showed that RA and RV were clustered separately, except for sample RA Late HF 3 that clustered separately as also observed in the principal component analysis plot (Fig 1A and 1B). For RV all control samples clustered together as did early and late HF, whereas samples of RA clustered alternating early and late stages of HF (Fig 1B). Clustering of the DEGs showed a clear separation of RA and RV and the different stages of HF (Fig 1C).

**Table 1:** Surface ECG parameters measured from control, early and late heart failure in dogs.

	N =	Heart/Body W Ratio [%]	Heart Rate [beat/min]	QRS [ms]	QT [ms]	QTc [ms]	P wave [mV]	PR [ms]
Control	9	0.80±0.13	116±18	57±5	182±7	252±14	0.37±0.11	90±14
Early HF	7–9	1.12±0.16 **	155±25***	68±6**	177±10*	282±13***	0.61±0.16***	106±13***
Late HF	5–8	1.13±0.13 **	144±13***	81±8***	178±5	275±16***	0.75±0.14***	120±13***

*p<0.05

**p<0.01 and

***p<0.001

**Fig 2:**
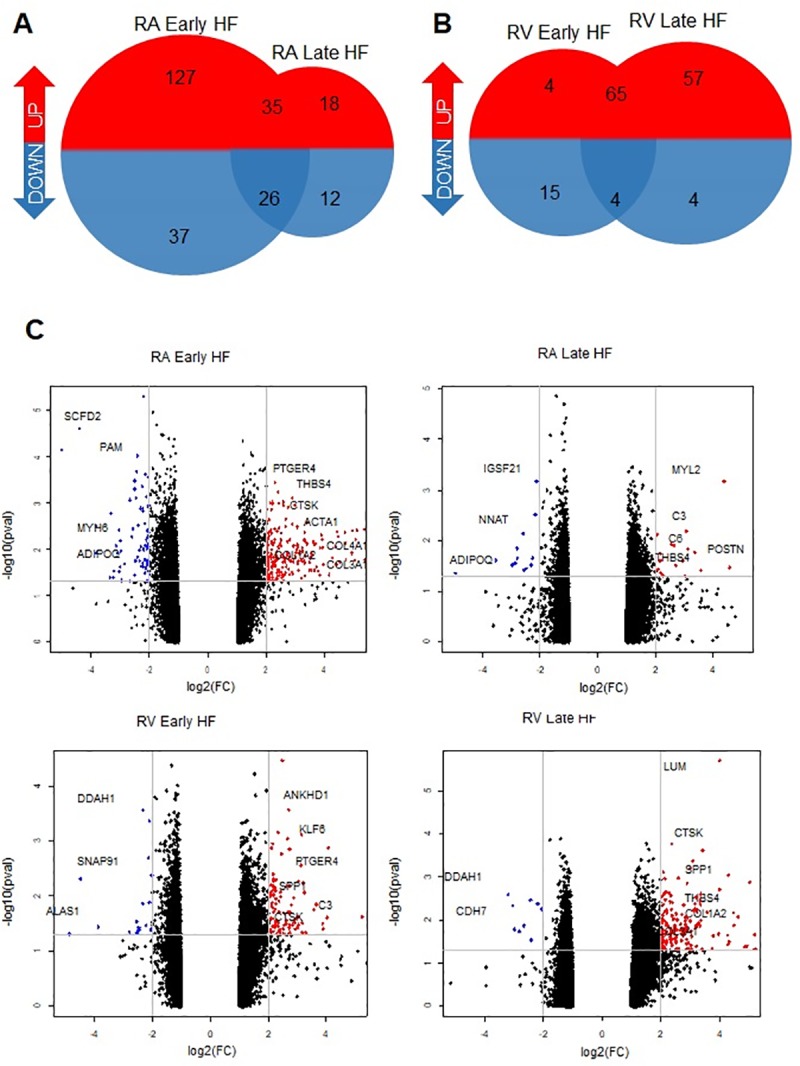
Comparison of differentially expressed genes (DEGs) between early and late stages of heart failure (HF). The Venn diagrams show the number of DEGs in each comparison and the number of upregulated (red) and downregulated (blue) overlapping genes between the early and late stages of HF in a canine model for (A) RA and (B) RV. All samples are compared to their respective control. (C) The Volcano plots show the total gene expression with positive (red) and negative (blue) log2 fold difference ≥2 (x-axis) against adjusted P-value ≤0.05 (y-axis). All other genes with adjusted P-value >0.05 are indicated in black.

Using a log2 fold difference ≥2 in combination with a significance threshold of P-adjusted value of 0.05, we identified 534 DEGs. Early stages of heart failure showed a higher number of DEGs in atria compared to ventricles (225 vs 88) and when compared to late stages in RA (225 vs 91) (Fig 2A). The RV showed more DEGs at late stages of HF (130 vs 88) when compared to early stages and RA at late HF (130 vs 91) (Fig 2B). Overall, the overlapping genes observed in RA included DEGs related to the gene ontology (GO) terms for complement activation (C3, C3, CFI), collagen metabolic process (*COL1A1*, *COL1A2*, *COL4A1*, *COL8A1 CTSK*) and circulatory system (*MYL2*, *MYL3*, *MLK*, *NPPB*, *FN1*). In the RV, the overlapping DEGs corresponded to the GO terms for extracellular matrix (*CTSK*, *CTSS*, *THBS4*, *SPP1*, *POSTN*), heart contraction (*NPPB*, *MYL1*, *MYL4*, *NPPA*) and cardiac muscle tissue (*ANKRD1*, *TNNT1 NPPA*).

The volcano plots display the DEGs, with selected transcripts highlighted based on the results obtained (Fig 2C).

### Selected GO terms show fibrotic and wound healing genes enrichment

**Fig 3:**
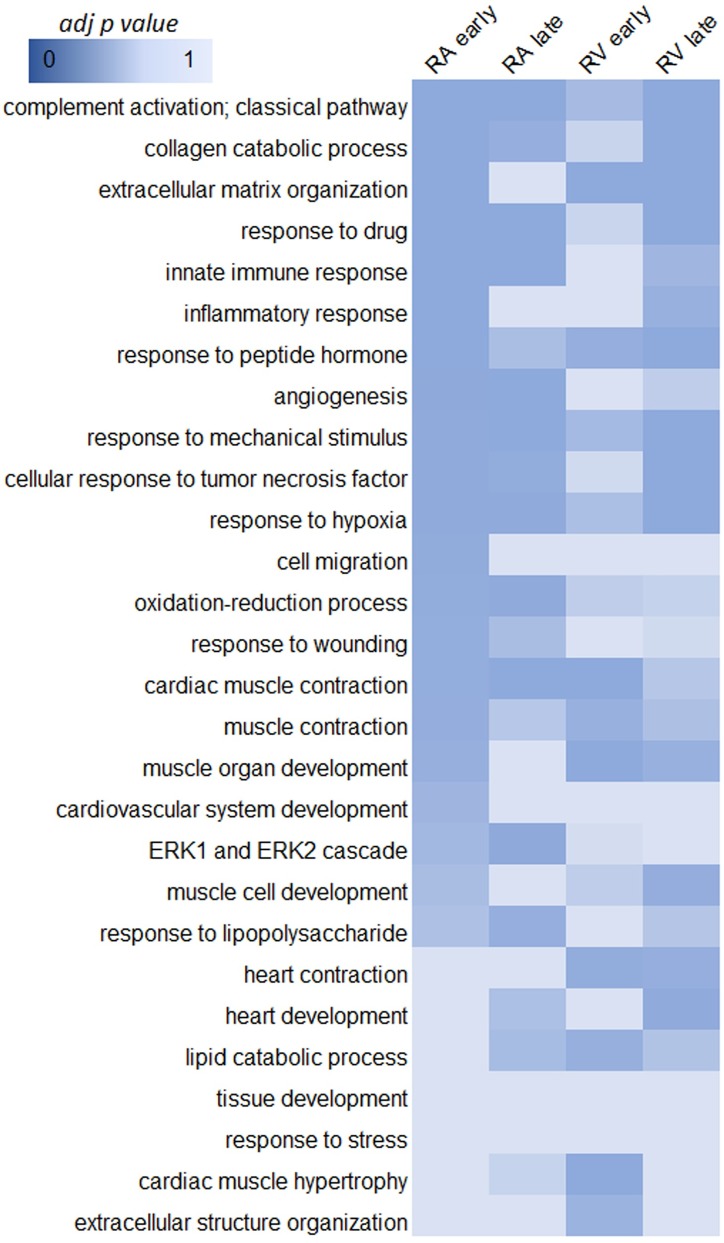
Enriched gene ontology (GO) terms belonging to biological processes. Enriched GO terms upregulated differentially expressed genes for comparisons between early and late stages of HF. Significantly enriched GO terms (Fisher's exact test; adjusted P-value ≤0.01) are shown in dark blue according to the color key.

To explore the functional characteristics of the DEGs, we performed GO analysis using iPathway Guide (Fig 3 and S1–S3 Tables). The heat map displays the GO terms with adjusted P-value ≤0.01. A marked difference between early and late stages of HF, as well as RA and RV, was observed. A higher number of biological processes are upregulated during the early stages of HF in RA and late stages of HF in RV, while late HF in RA and early HF in RV showed a lower number of upregulated biological processes. No enrichment of GO terms was found for downregulated genes, except for the response to steroid hormones at early stages of HF in the RV. Among the most enriched processes we found were collagen catabolic process, extracellular matrix organization, immune response, inflammatory response and response to wounding. These results suggest a process of active remodeling such as fibrosis. These changes are not present in RA but appear in RV at late stages of HF. Interestingly, the late stages of HF in RA show immune and inflammatory response processes, which are not present in RV (Fig 3).

### Microarray results contain relevant biological information

**Fig 4:**
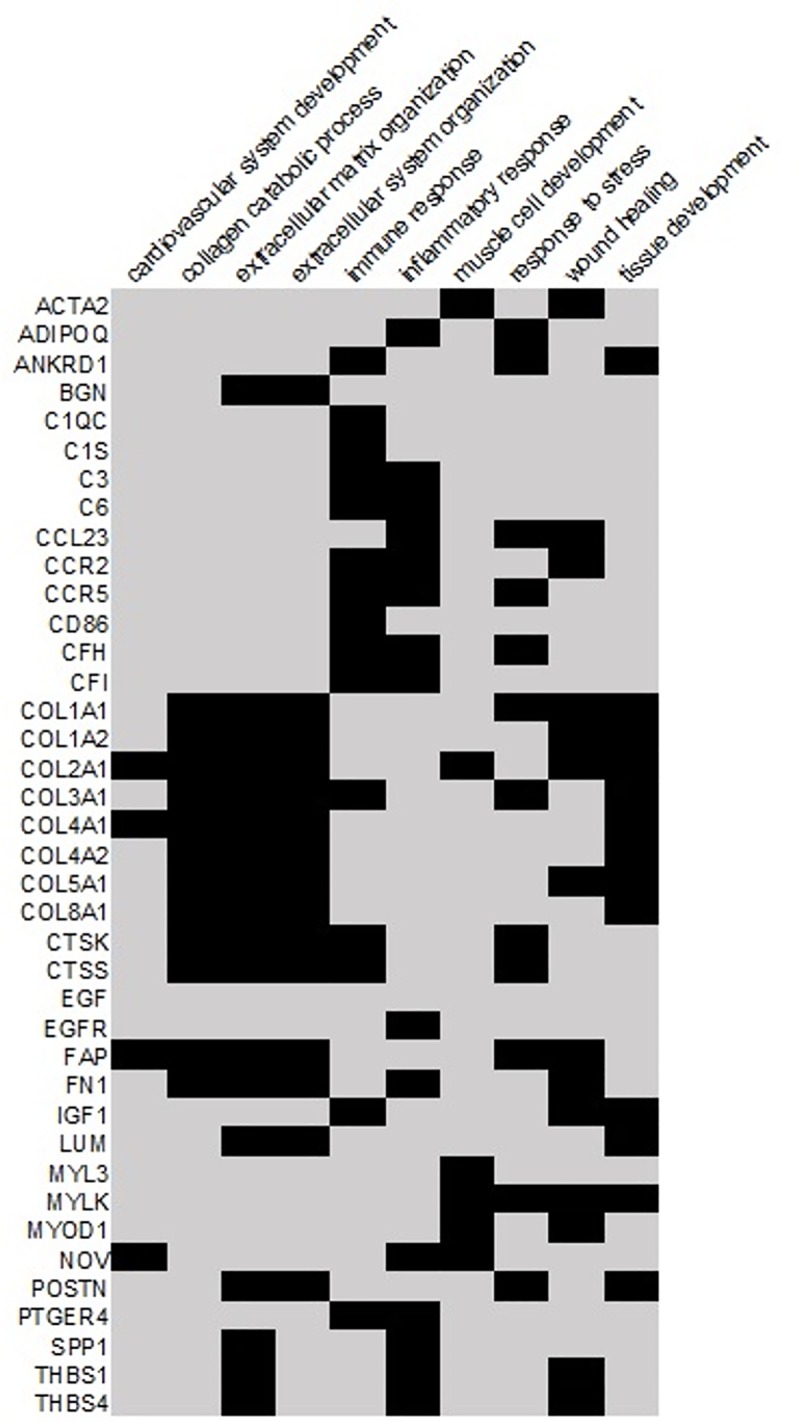
Several genes participate in more than one biological process. Heat map of top scored signaling pathways enriched in heart failure microarray analysis. Every row represents a gene, and every column represents a pathway. Top significant signaling pathways for commonly enriched genes are presented on the heat map in black. Pathway enrichment analysis was done using Advaita iPathway Guide, and full list of pathways can be found in S1–S3 Tables. Only genes that have fold change above two were presented in the heat map.

Among the top 50 pathways significantly associated with our dataset (S1 Table) we found many known to play a significant role in heart failure, fibrosis and inflammatory response. We performed a cluster analysis of transcripts that had a fold change above two and were present in at least one pathway. Fig 4 represents a summary of upregulated transcripts in the following 10 most overrepresented pathways: cardiovascular system development, collagen catabolic process, extracellular matrix organization, extracellular system organization, immune response, inflammatory response, muscle cell development, response to stress, wound healing and tissue development. We identified several transcripts that overlap in many of these processes. Genes such as *COL1A1*, *COL1A2*, *COL2A1*, *COL3A1*, *COL4A1*, *COL4A2*, *COL5A1*, *COL8A1*, *FAP* and *FN1* are involved in collagen and extracellular matrix processes as well as heart development and wound healing (Fig 4). Genes like *C1QC*, *C1S*, *C3*, *CS*, *CCL23*, *CCR2*, *CCR5*, *CD86*, *CFH* and *CFI* overlap in immune response and inflammatory response processes. Pathways analysis supports the transcriptomic profiling in early and late stages of HF (S1–S4 Figs).

### Real-time RT-PCR validates DEGs detected in microarray

**Fig 5:**
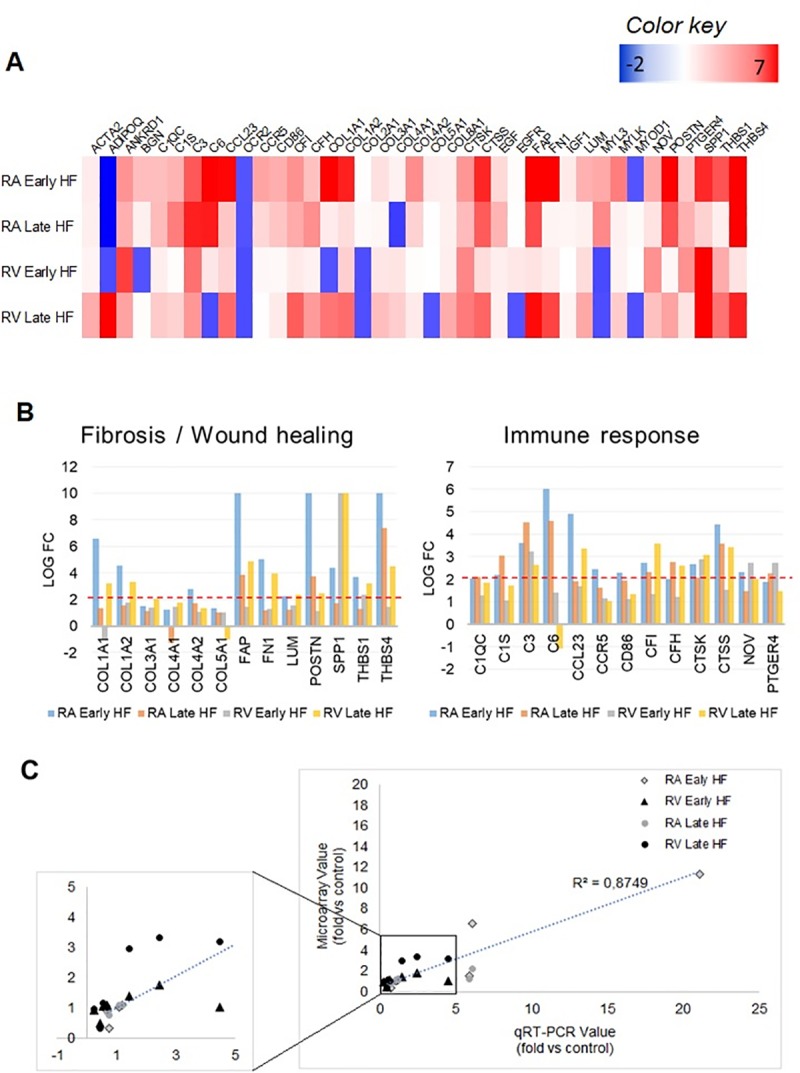
RT-qPCR validates the differentially expressed genes (DEGs) identified in microarray analysis. (A) Heat map of genes differentially expressed in heart failure vs. control dogs (ANOVA, p<0.05, fold change >2 or <−2, n = 3). Upregulated gene expression is displayed in red and downregulated gene expression in blue. Genes are presented in alphabetical order. (B) Relative expression of DEGs associated with fibrosis/wound healing and immune response. Mean fold change with cut-off filter criteria of at least a 2-fold change and an adjusted P-value <0.05 was used to identify upregulation. (C) Correlation between microarray expression and qPCR for selected genes. Expression levels normalized to GAPDH (n = 5). Data presented as fold change relative to control levels. One-way ANOVA with Bonferroni post hoc analysis.

We analyzed the expression of the main DEGs involved in the top 10 overrepresented biological processes from Fig 4. The heat map shows the upregulated (red) and downregulated (blue) transcripts in RA and RV at early and late stages of HF (Fig 5A). As expected, a greater number of upregulated genes are observed in RA during early HF and RV during late HF. These genes are involved mainly in fibrosis and wound healing as well as immune response and inflammation (Fig 5B). We found that *COL1A1*, *COL1A2*, *FAP*, *FN1*, *LUM*, *POSTN*, *SPP1*, *THBS1* and *THBS4* are highly upregulated in early HF in RA and late HF in RV, suggesting that the fibrotic process occurs earlier in RA. Genes involved in inflammatory processes such as *C1QC*, *C1S*, *C3*, *C6*, *CCL23*, *CCR5*, *CD86*, *CFI*, *CTSK*, *CTSS*, *NOV* and *PTGER4* show a similar trend, but in this case, they are present in late stages of HF in RA.

Quantitative real-time RT-PCR was performed on 7 randomly selected genes for RA and RV in control and HF dogs. Genes identified involved in fibrosis processes were selected to represent upregulated, downregulated or non-significantly-changed DEGs. Fig 5C compares genes expression levels by real-time RT-PCR with microarray results, which showed a good correlation (R^2^ = 0.874). Thus, there was good agreement overall.

## Discussion

In this study, utilizing tissues from our previously characterized canine VTP model of HF [5], we compare atrial versus ventricular remodeling via mRNA expression changes in early (2–3 weeks of VTP) and late (5–6 weeks of VTP) stages of HF in dogs. The enriched biological processes point to marked differences in the atrial versus ventricular response and time-dependent evolution of fibrotic changes in both chambers. These results are consistent with the changes observed in cardiac dimensions, hemodynamic, electrophysiological parameters histological changes as well as the development of both atrial and ventricular arrhythmia previously reported by our group in these same animals [5].

Several studies have reported substantial differences in gene expression between atria and ventricles of mice, dogs and humans [16–19]. Previous studies by Barth et al showed upregulation of apoptotic and signal transduction genes in RA with a “ventricularization” of the atrial tissues [17]. Our results show consistent differences between right atrial and right ventricular responses, with changes in the right atria being faster, larger and more transient than in the ventricle. Structural remodeling in atria during HF has great significance for the pathophysiology of AF [20]. We observed prominent remodeling in VTP-induced HF, where it plays a central role in development of the substrate for AF and atrial conduction impairment. GO-based analysis demonstrated that genes upregulated in RA during early HF are significantly associated with extracellular matrix pathways, immune response, inflammatory response and response to wounds, suggesting a developing fibrotic process. In late HF, the RA showed upregulation of genes involved in complement activation, immune response, ERK1 and ERK2 cascade, and response to hypoxia, suggesting an apoptotic state after the fibrotic process has ended apoptosis ensues. Although we did not perform studies specifically designed to quantify the extent to which inflammation developed in our preparations, previous studies have provided evidence for the development of an inflammatory process in the VTP-model of heart failure employed in our study [7,21,22]. The transcriptomic changes observed in the present study are consistent with these previous reports.

The right ventricle showed a different profile to that observed in the atrium. During early HF, the RV showed upregulation of genes related to cardiac muscle contraction and muscle organ development, without any indication of fibrosis. During late stages of HF, the RV showed upregulation of collagen catabolic process, extracellular matrix organization, response to hypoxia and response to tumor necrosis factor. These results suggest that the remodeling in the RV occurs later, and the fibrosis arises during late HF.

The upregulated expression of fibrosis-associated markers in early but not late stages of HF in RA, suggests an inhibitory feedback loop.

Our results are consistent with previous studies by Hanna et al who performed a similar analysis of the left heart. In that study, LA showed much stronger inflammatory and apoptotic changes than the LV [22].

The microarray data analyzed in this study confirms the presence of an “AF-vulnerable window” as previously reported [5]. The susceptibility to AF was increased in early HF and less so in late HF, whereas the susceptibility to VT/VF increased in late but not early HF. These atrioventricular distinctions may have important implications for our understanding of the mechanisms underlying HF remodeling and for the development of new approaches to therapy of AF.

## Clinical implications

The present study advances our understanding of the transcriptional basis for the development of structural remodeling that occurs during the progression of HF. Significant fibrosis-associated transcriptional changes in RA in early HF and in RV in late HF are observed in an experimental model of HF known to recapitulate the fundamental mechanical, electrophysiological, biochemical and molecular changes observed in clinical HF. The early HF remodeling is much more prominent in RA with enrichment of extracellular matrix, circulatory system, wound healing and immune response pathways; In contrast, RV showed no signs of remodeling in early HF but enrichment of extracellular matrix and wound healing in late HF. These observations offer important insights into the development of structural remodeling associated with clinical HF as well as insights into novel approaches to therapy.

The transcriptomic alterations in gene expression observed in the present study are consistent with the development of arrhythmogenesis in atria in early but not late HF and in the ventricles in late but not early HF as reported in an experimental model of HF. Atrial Fibrillation (AF) affects a large fraction of patients with chronic heart failure (HF) and is associated with poor long-term prognosis. Although AF is commonly associated with atrial structural remodeling (ASR), principally characterized by atrial dilatation and fibrosis, the occurrence of AF in the full spectrum of ASR is poorly defined. Clinical studies of patients with HF suggest the possibility that advanced ASR is associated with a less frequent AF occurrence than moderate ASR. These observations highlight the need for clinical studies aimed at providing a direct test of the hypothesis that severe fibrosis is associated with a lower burden of AF than moderate fibrosis in patients with HF and the implications of therapeutic interventions designed to reverse remodel the atria.

## Limitations

Although the canine VTP-induced HF model recapitulates many clinical features of HF and HF-induced AF, it represents a rapidly developing, non-ischemic, dilated cardiomyopathy that becomes terminal in a period of several weeks. This time-course is much faster than that of many clinical forms of HF in humans, which often develops over months or years. This limitation would apply to most animal models of HF and needs to be taken into consideration when extrapolating these results to clinical contexts. Our study was designed to focus on the temporal window of vulnerability for AF and VF observed during the first 6 weeks of ventricular pacing (VTP)-induced heart failure (HF). The development of fibrosis in the ventricles clearly lags behind that of the atrium and it is possible that the two would be comparable had we extended the study beyond 6 weeks. Additional studies are needed to test this hypothesis.

Although this study has focused mainly in the transcriptome analysis of HF in a canine model, protein translation analysis has been challenging due to the unavailability of canine validated antibodies. Our group is currently working on this in a separate study using a different HF model.

Another limitation to take into consideration is the small sample size used in this study (n = 3) due to both the high costs and the long periods of time that takes to complete the studies.

The microarray-based transcriptome analysis is a well-validated tool for global transcriptomic analysis. In the current study involving canine tissues, microarray is more informative, reliable and conclusive than the NextGen sequencing platform, which in other cases could be argued to provide a more complete analysis of the transcriptome. The sequencing and annotation of the canine genome is still evolving, as recently highlighted by Parker and co-workers [23]. It is also noteworthy that the canine genome was fundamentally incomplete when this investigation was initiated.

## Supporting information

S1 Fig Enriched extracellular matrix receptor interaction pathway for early heart failure in RA. The pathway diagram is overlaid with the fold change values of each gene. The highest positive fold change is shown in dark red. The legend describes the values on the gradient. For each gene family, the color corresponding to the gene with the highest absolute fold change is displayed.
(TIF)

S2 Fig Enriched complement cascade pathway for late heart failure in RA. The pathway diagram is overlaid with the fold change values of each gene. The highest positive fold change is shown in dark red. The legend describes the values on the gradient. For each gene family, the color corresponding to the gene with the highest absolute fold change is displayed.
(TIF)

S3 Fig Enriched extracellular matrix receptor interaction pathway for early heart failure in RV. The pathway diagram is overlaid with the fold change values of each gene. The highest positive fold change is shown in dark red. The legend describes the values on the gradient. For each gene family, the color corresponding to the gene with the highest absolute fold change is displayed.
(TIF)

S4 Fig Enriched extracellular matrix receptor interaction pathway for late heart failure in RV. The pathway diagram is overlaid with the fold change values of each gene. The highest positive fold change is shown in dark red. The legend describes the values on the gradient. For each gene family, the color corresponding to the gene with the highest absolute fold change is displayed.
(TIF)

S1 Table Top identified biological processes. Only the top scoring biological processes are described.
(PDF)

S2 Table Top identified molecular functions. Only the top scoring functions are described in the table below.
(PDF)

S3 Table Top identified cellular components. Only the top scoring cellular components are described.
(PDF)
