# Supplementary figures and images for: Transcriptional changes associated with advancing stages of heart failure underlie atrial and ventricular arrhythmogenesis
Source: PLoS One. 2019 May 13;14(5):e0216928. doi: 10.1371/journal.pone.0216928 (PMC6513089; doi:10.1371/journal.pone.0216928)

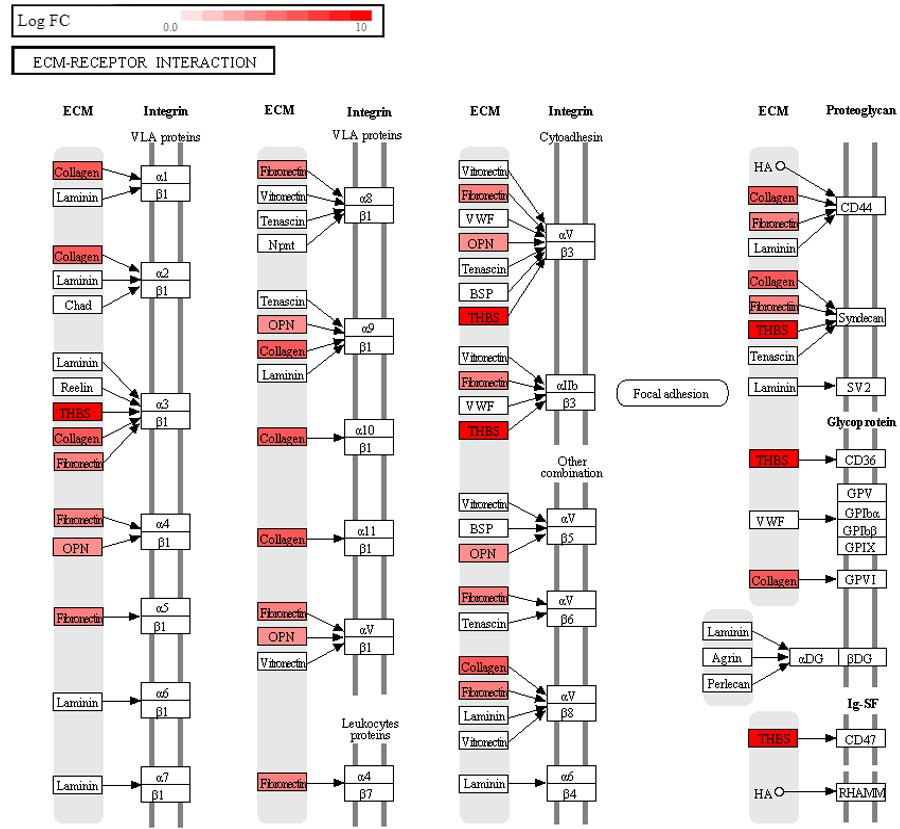

Supplement: S1 Fig — The pathway diagram is overlaid with the fold change values of each gene. The highest positive fold change is shown in dark red. The legend describes the values on the gradient. For each gene family, the color corresponding to the gene with the highest absolute fold change is displayed. (TIF) [file pone.0216928.s001.tif]

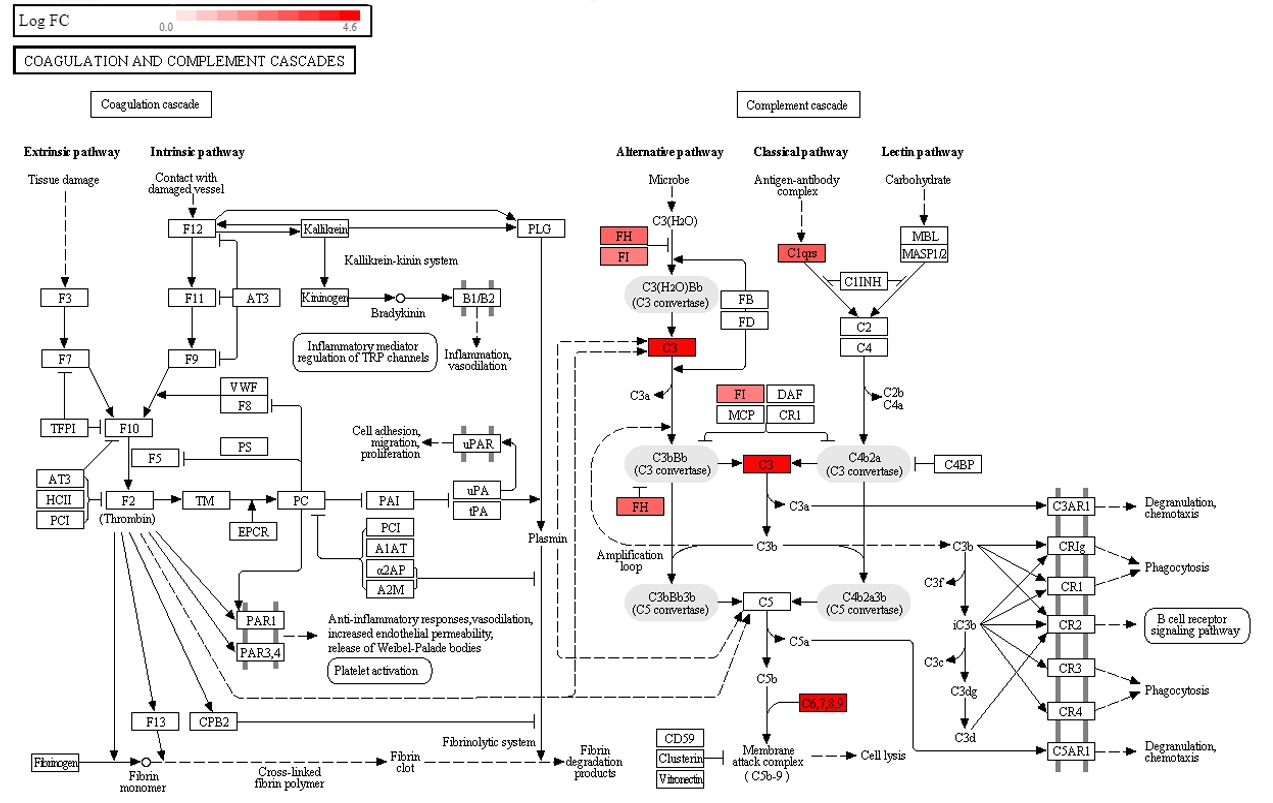

Supplement: S2 Fig — The pathway diagram is overlaid with the fold change values of each gene. The highest positive fold change is shown in dark red. The legend describes the values on the gradient. For each gene family, the color corresponding to the gene with the highest absolute fold change is displayed. (TIF) [file pone.0216928.s002.tif]

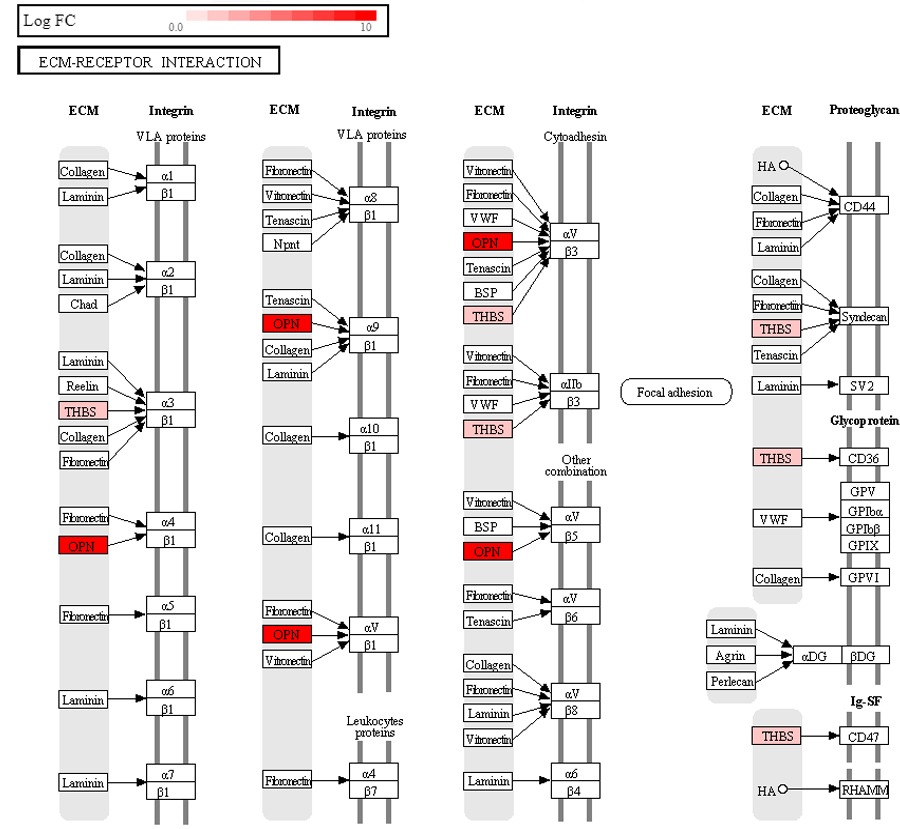

Supplement: S3 Fig — The pathway diagram is overlaid with the fold change values of each gene. The highest positive fold change is shown in dark red. The legend describes the values on the gradient. For each gene family, the color corresponding to the gene with the highest absolute fold change is displayed. (TIF) [file pone.0216928.s003.tif]

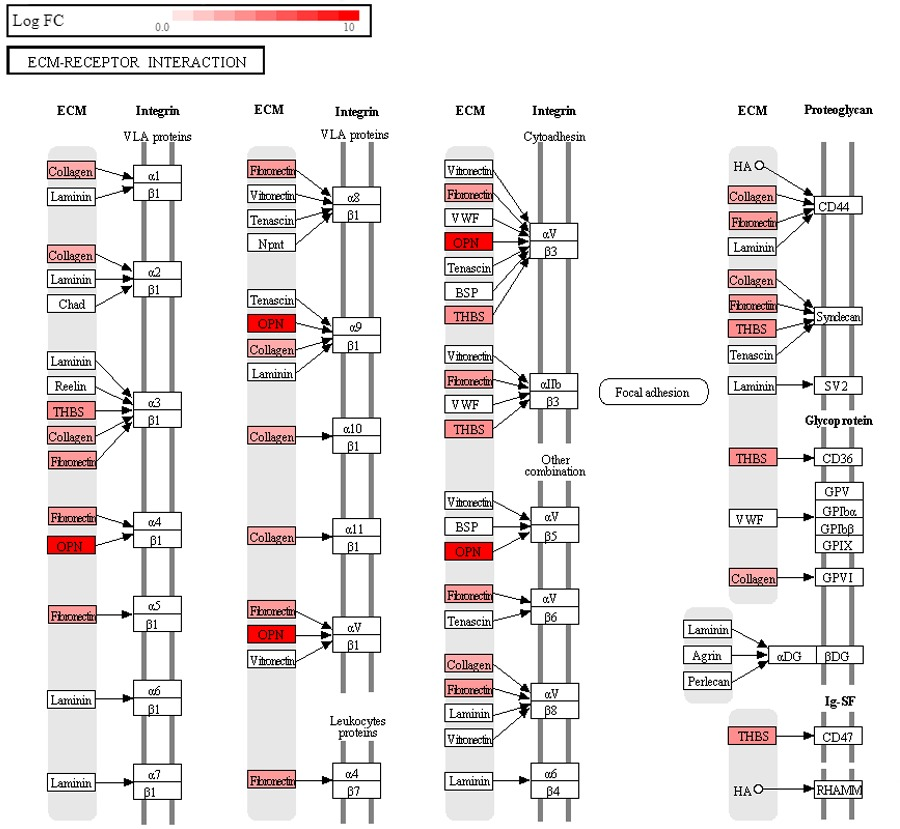

Supplement: S4 Fig — The pathway diagram is overlaid with the fold change values of each gene. The highest positive fold change is shown in dark red. The legend describes the values on the gradient. For each gene family, the color corresponding to the gene with the highest absolute fold change is displayed. (TIF) [file pone.0216928.s004.tif]
