# Supplementary material for: Transcriptional changes associated with advancing stages of heart failure underlie atrial and ventricular arrhythmogenesis
Source: PLoS One. 2019 May 13;14(5):e0216928. doi: 10.1371/journal.pone.0216928 (PMC6513089; doi:10.1371/journal.pone.0216928)
Supplement: S1 Table — Only the top scoring biological processes are described. (PDF) [file pone.0216928.s005.pdf]

S1 Table. Top identified biological processes. Only the top scoring biological processes are described.

GO	goName	DEGs	All Genes	pv_elim
RA Early				
GO:0030198	extracellular matrix organization	22	309	0,000075
GO:0042493	response to drug	15	363	0,00025
GO:0045087	innate immune response	21	592	0,00033
GO:0006954	inflammatory response	19	553	0,00086
GO:0043434	response to peptide hormone	12	365	0,00366
GO:0001525	angiogenesis	19	389	0,00483
GO:0016477	cell migration	29	1086	0,0136
GO:0009611	response to wounding	19	551	0,02461
GO:0006936	muscle contraction	11	305	0,02823
RA Late				
GO:0006958	complement activation; classical pathway	5	23	7,20E-08
GO:0045087	innate immune response	14	592	0,00046
GO:0009612	response to mechanical stimulus	6	183	0,00239
GO:0001525	angiogenesis	11	389	0,00273
GO:0042493	response to drug	9	363	0,00273
GO:0070371	ERK1 and ERK2 cascade	5	230	0,0051
GO:0055114	oxidation-reduction process	14	841	0,00604
GO:0001666	response to hypoxia	6	258	0,00712
GO:0032496	response to lipopolysaccharide	5	265	0,0331
RV Early				
GO:0060048	cardiac muscle contraction	4	107	0,00189
GO:0007517	muscle organ development	8	324	0,00302

GO:0050729	positive regulation of inflammatory response	4	102	0,0126
GO:0060047	heart contraction	7	235	0,01848
GO:0016042	lipid catabolic process	4	258	0,03814
GO:0006936	muscle contraction	8	305	0,0472
GO:0008016	regulation of heart contraction	5	208	0,05645
GO:0030198	extracellular matrix organization	5	309	0,05988
GO:0043062	extracellular structure organization	5	310	0,06046
RV Late				
GO:0030574	collagen catabolic process	7	61	7,7E-06
GO:0009612	response to mechanical stimulus	10	183	0,000019
GO:0071560	cellular response to transforming growth factor b stimulus	9	194	0,00027
GO:0030198	extracellular matrix organization	20	309	0,00042
GO:0042493	response to drug	14	363	0,00057
GO:0001666	response to hypoxia	9	258	0,00102
GO:0071356	cellular response to tumor necrosis factor	7	221	0,0022
GO:0043434	response to peptide hormone	11	365	0,00227
GO:0007507	heart development	9	462	0,01317
