# Supplementary material for: Transcriptional changes associated with advancing stages of heart failure underlie atrial and ventricular arrhythmogenesis
Source: PLoS One. 2019 May 13;14(5):e0216928. doi: 10.1371/journal.pone.0216928 (PMC6513089; doi:10.1371/journal.pone.0216928)
Supplement: S2 Table — Only the top scoring functions are described in the table below. (PDF) [file pone.0216928.s006.pdf]

S2 Table. Top identified molecular functions. Only the top scoring functions are described in the table below.

GO	goName	DEGs	All Genes	pv_elim
RA Early				
GO:0008201	heparin binding	13	144	3,10E-09
GO:0001968	fibronectin binding	5	21	0,000002
GO:0004252	serine-type endopeptidase activity	10	145	2,7E-06
GO:0005509	calcium ion binding	18	585	0,000035
GO:0005201	extracellular matrix structural constituent	8	72	0,000073
GO:0005178	integrin binding	7	105	0,00011
GO:0005518	collagen binding	5	60	0,00299
GO:0008083	growth factor activity	6	148	0,00447
GO:0042803	protein homodimerization activity	13	634	0,01476
RA Late				
GO:0004252	serine-type endopeptidase activity	7	145	5,9E-06
GO:0008201	heparin binding	6	144	0,000065
GO:0001968	fibronectin binding	3	21	0,00013
GO:0043394	proteoglycan binding	3	29	0,00036
GO:0008307	structural constituent of muscle	3	39	0,00086
GO:0008083	growth factor activity	4	148	0,00559
GO:0005178	integrin binding	3	105	0,01409
GO:0005509	calcium ion binding	7	585	0,0219
GO:0042803	protein homodimerization activity	7	635	0,03236
RV Early				
GO:0001968	fibronectin binding	3	21	0,00011
GO:0008307	structural constituent of muscle	3	39	0,00071

GO:0003779	actin binding	7	366	0,00125
GO:0042805	actinin binding	3	28	0,00316
GO:0005200	structural constituent of cytoskeleton	3	87	0,00709
GO:0044325	ion channel binding	3	105	0,01184
GO:0008201	heparin binding	3	144	0,02721
GO:0005518	collagen binding	3	60	0,02732
GO:0008022	protein C-terminus binding	3	170	0,04139
RV Late				
GO:0008201	heparin binding	16	144	1,10E-14
GO:0005201	extracellular matrix structural constituent	9	72	4,40E-08
GO:0005509	calcium ion binding	19	585	7,00E-08
GO:0005518	collagen binding	8	60	2,40E-07
GO:0005178	integrin binding	6	105	0,00015
GO:0004252	serine-type endopeptidase activity	6	145	0,00082
GO:0042802	identical protein binding	16	928	0,00163
GO:0046983	protein dimerization activity	12	962	0,06058
GO:0050839	cell adhesion molecule binding	11	423	0,07956
