# Supplementary material for: Transcriptional changes associated with advancing stages of heart failure underlie atrial and ventricular arrhythmogenesis
Source: PLoS One. 2019 May 13;14(5):e0216928. doi: 10.1371/journal.pone.0216928 (PMC6513089; doi:10.1371/journal.pone.0216928)
Supplement: S3 Table — Only the top scoring cellular components are described. (PDF) [file pone.0216928.s007.pdf]

S3 Table. Top identified cellular components. Only the top scoring cellular components are described.

GO	goName	DEGs	All Genes	pv_elim
RA Early				
GO:0005615	extracellular space	47	1090	5,90E-14
GO:0070062	extracellular exosome	48	2334	3,40E-07
GO:0005604	basement membrane	10	85	0,000011
GO:0005578	proteinaceous extracellular matrix	25	330	0,000051
GO:0009986	cell surface	19	623	0,00013
GO:0005581	collagen trimer	11	77	0,0004
GO:0005783	endoplasmic reticulum	32	1451	0,00456
GO:0031012	extracellular matrix	30	470	0,0068
GO:0098552	side of membrane	10	361	0,02532
RA Late				
GO:0005615	extracellular space	22	1090	1,1E-06
GO:0070062	extracellular exosome	20	2338	0,0047
GO:0044297	cell body	6	432	0,0163
GO:0030017	sarcomere	5	168	0,0239
GO:0000323	lytic vacuole	7	477	0,0665
GO:0005764	lysosome	7	477	0,0665
GO:0031012	extracellular matrix	5	470	0,0715
GO:0009986	cell surface	6	627	0,0758
GO:0030016	myofibril	6	186	0,077
RV Early				
GO:0030018	Z disc	6	101	0,000008
GO:0030017	sarcomere	11	168	0,0011

GO:0015629	actin cytoskeleton	9	426	0,0055
GO:0031674	I band	7	112	0,0468
GO:0005615	extracellular space	10	1090	0,0629
GO:0030016	myofibril	12	186	0,0699
GO:0031012	extracellular matrix	5	470	0,0715
GO:0005739	mitochondrion	11	1460	0,0806
GO:0005924	cell-substrate adherens junction	5	371	0,0927
RV Late				
GO:0005615	extracellular space	47	1090	8,40E-18
GO:0070062	extracellular exosome	44	2338	3,00E-09
GO:0005604	basement membrane	9	85	2,50E-07
GO:0005578	proteinaceous extracellular matrix	24	330	0,000023
GO:0031012	extracellular matrix	30	470	0,00023
GO:0005783	endoplasmic reticulum	26	1452	0,00123
GO:0030016	myofibril	10	186	0,00739
GO:0031988	membrane-bounded vesicle	55	3053	0,01229
GO:0005581	collagen trimer	7	77	0,01716
